# Evaluating Emergency Department Utilization among Undocumented Patients Receiving Care at a Community Health Clinic

**DOI:** 10.1007/s10903-025-01723-9

**Published:** 2025-07-10

**Authors:** Ana Acevedo, Elizabeth Whidden, Francisco Zepeda, K. Jane Muir, D. Daphne Owen

**Affiliations:** 1 University of Pennsylvania, Philadelphia, USA; 2 University of California, Berkeley, Berkeley, USA

**Keywords:** Immigrant Health, Emergency Department Utilization, Undocumented Health, Community Health Clinics

## Abstract

Undocumented immigrants in the US rely on community health clinics (CHCs) and emergency departments (EDs) for preventive and acute care due to health insurance ineligibility and financial constraints. CHCs provide low-cost, accessible healthcare for thousands of undocumented immigrants in cities throughout the U.S. Limited evidence exists on this population’s use of EDs despite establishing care in CHCs. This cross-sectional study described the key characteristics of ED visits among undocumented immigrants with established care at a single CHC in a major US city. ED visits (*n* = 319) were extracted using a random sample of patient records from a CHC serving a largely undocumented population between April 1, 2023, and April 1, 2024. The most common discharge diagnoses were categorized as infections (23%), injuries (22%), gastrointestinal diseases (16%), and OB/GYN diseases (11%). Disposition data showed that 81% of patients were discharged directly from the ED, 14% were admitted to the hospital, and 4% were placed under observation. For 309 visits, 61% were classified as preventable by the NYU ED algorithm (NYU-EDA). These findings suggest that most visits were preventable or primary care treatable, often driven by acute health issues rather than chronic care needs. Study results inform how CHCs, EDs, and health systems can address care gaps, enhance preventative care strategies, and advance equitable care delivery.

**Supplementary Information:**

The online version contains supplementary material available at 10.1007/s10903-025-01723-9.

## Introduction

The estimated twelve million undocumented individuals residing in the United States face significant barriers to consistent healthcare access [1]. A 2023 nationally representative survey of immigrants in the U.S. found that 50% of undocumented immigrant adults reported being uninsured compared to just 6% of naturalized citizens [2]. When undocumented individuals do seek care, limited interpreter resources within health systems contribute to delays in access, poor treatment adherence, and lower use of screening services [3]. Additionally, many undocumented immigrants refrain from seeking healthcare altogether due to fear of deportation and discrimination [4]. These insurance disparities, language concerns, and legal obstacles result in this population receiving less primary and specialty care and experiencing higher rates of untreated medical conditions​ [5].

Emergency departments (EDs) are often the de facto entry point for uninsured and undocumented individuals to the healthcare system, as the Emergency Medical Treatment & Labor Act (EMTALA) mandates that hospitals provide ED care to all presenting patients regardless of insurance status [6]. While EDs are community-facing, increased ED utilization fragments high-quality care coordination and continuity for preventative services typically provided in primary care settings. This results in inefficient use of medical resources, higher costs, and suboptimal management of both acute and chronic conditions. Moreover, studies have shown that access to consistent primary care can significantly reduce avoidable ED visits among undocumented immigrants and improve disease management to likely enhance long-term health outcomes​ [7]​.

Community health clinics (CHCs) are one of the few sources of primary care for undocumented and uninsured immigrants, offering an alternative to higher acuity care settings. Many CHCs provide low-cost care to patients regardless of their insurance coverage or documentation status. However, CHCs do not routinely document immigration status, so there is limited data on the utilization of CHCs by undocumented immigrants. CHC electronic health records (EHRs) facilitate the implementation of quality improvement programs that can improve health outcomes for clinic patients. However, CHCs often lack data on their patients’ patterns of healthcare usage outside of their clinic, leading to challenges in designing interventions to reduce ED visits and hospitalizations for their patients.

The objective of this study was to describe the clinical characteristics of a subset of undocumented individuals in EDs. Given the healthcare access challenges faced by undocumented individuals, we hypothesized that a substantial proportion of their ED visits would involve non-emergent cases better suited for lower-acuity care settings and we aimed to quantify this proportion by linking EHRs from a CHC serving thousands of undocumented patients every year to ED records at a local academic health system, we report key characteristics of these ED visits.

## Methods

### Study Design

This study used a descriptive, cross-sectional design to examine ED visit outcomes among patients from a CHC in a major US city presenting to a local academic health system.

### Setting & Subjects

Patient encounters were extracted from a CHC that is a nationally recognized 501(c)(3) nonprofit health and wellness organization serving its city’s urban, undocumented immigrant community with an average of 8,000 to 10,000 patient visits annually. Patients were identified as undocumented during intake based on lack of health insurance and a Social Security number. Patients who had an encounter at the CHC documented between April 1, 2023, and April 1, 2024, were eligible for inclusion. A simple random sample of eligible patient records was selected for review, with the sample size determined by the practical constraints of manually extracting chart data within the study’s designated timeframe. Extracted records were matched with ED records from hospitals at a health system in the region, with available records spanning from 2011 to 2024. The records were deterministically matched using first name, last name, and date of birth. ED records from other local health systems were extracted using Epic Care Everywhere. Patients under 18 years of age or without a documented ED visit were excluded.

### Data Collection and Management

A list of eligible participants for this study was generated from the CHC EHR system. Patients randomly selected from this list were matched to corresponding records in the academic health system’s EHR. The data from these ED visits were manually abstracted by the investigators and subsequently entered and stored on RedCap, a secure web application for building and managing online databases. All patient data were deidentified to maintain confidentiality. This study was approved by the academic health system’s Institutional Review Board through expedited review. Consent for health record exchange was obtained from the CHC and academic health system under a formal Data Usage Agreement.

### Sample Selection

**Fig. 1:**
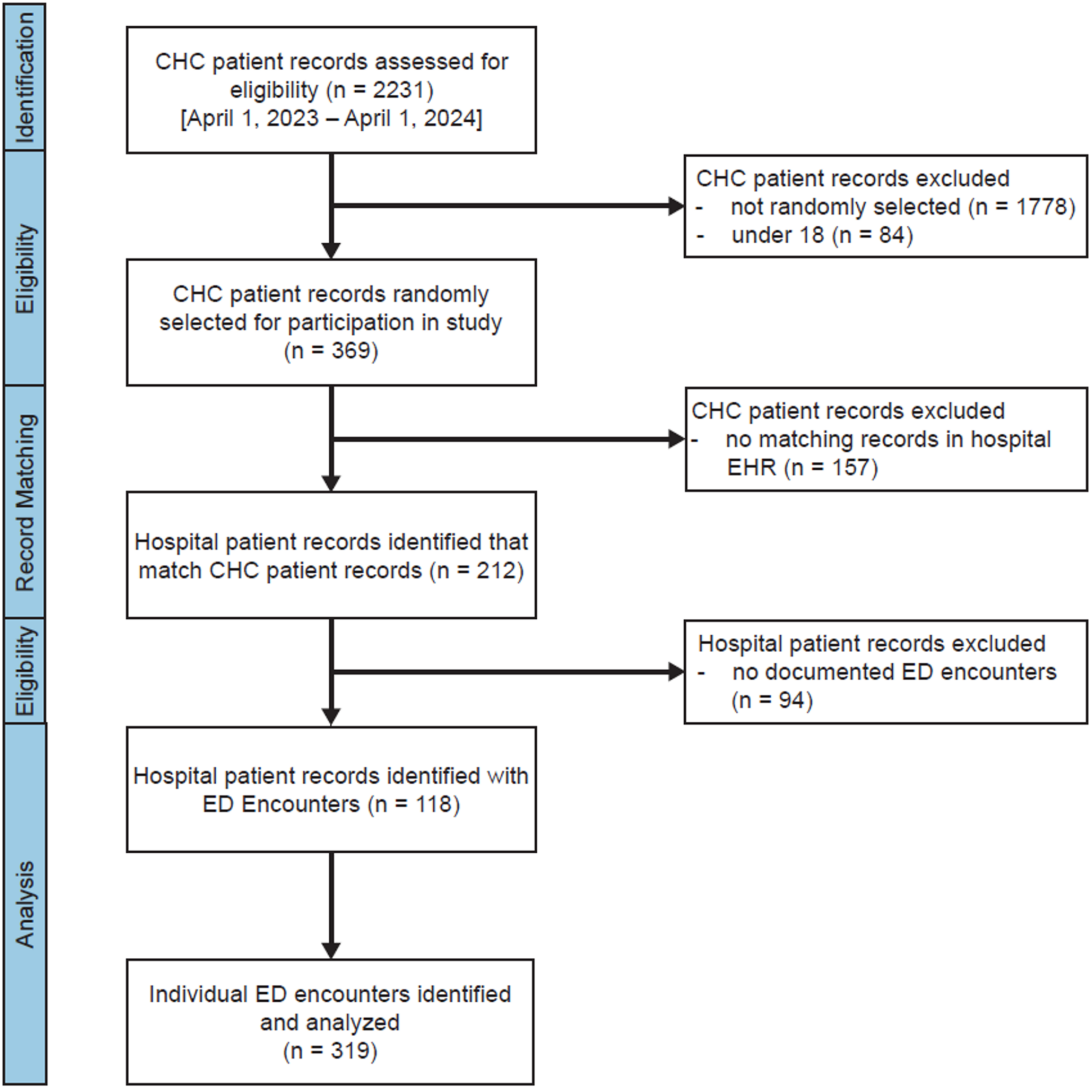
Study flow diagram. This figure illustrates the flow of participants through the study. Patients were first identified using electronic health records from the community health clinic (CHC). A simple random sample of all patients over 18 years old was selected for inclusion. A subset of these patients were found to have matching records in the academic health system’s EHR. These records were then reviewed for eligible ED encounters to be included in the analysis

A total of 2,231 CHC records from patients with visits to the CHC between April 1, 2023, and April 1, 2024, were assessed for eligibility. Of these, 84 (3%) were excluded because the patients were under 18 years old (Fig. 1). From the remaining 2,147 records, 369 patients (17%) were randomly selected for inclusion. Among them, 157 (42%) were excluded due to a lack of matching records in the academic health system’s EHR, resulting in 212 matched patient records. Of these, 94 (44%) were excluded for having no documented ED encounters, yielding a final sample of 118 academic health system records with at least one ED encounter.

### Measures

#### Demographics

Patient demographic variables chosen for abstraction included age, sex, race, ethnicity, and insurance status as determined and documented by ED registrars. For each ED visit, the chief concern, discharge diagnosis, Emergency Severity Index (ESI) acuity level, and disposition were abstracted.

#### Chief Concerns

Chief concerns, defined as the primary reason for the patient’s visit as stated at triage, were categorized based on symptom-based or organ system classifications. Categories were adapted from the Centers for Medicare & Medicaid Services (CMS) latest Major Diagnostic Category (MDC) classifications [8]. These categories included: constitutional, genitourinary, gastrointestinal OB/GYN, chest pain, respiratory, rash, ear, nose, and throat (ENT), psychiatric, lab testing, neurologic, ophthalmologic, neurologic, and other. The constitutional category encompasses generalized symptoms such as fatigue, fever, weight loss, or malaise that do not localize to a single organ system. Genitourinary concerns involve issues related to the urinary or reproductive systems, such as dysuria, hematuria, incontinence, or testicular pain. ENT concerns included infections, hearing loss, nosebleeds, or throat pain not primarily classified under respiratory conditions. Psychiatric concerns covered mental health-related concerns such as anxiety, depression, suicidal ideation, or altered mental status. Lab testing referred to visits primarily for routine blood work, STI screening, or follow-up testing, rather than acute symptomatic concerns. Neurologic concerns encompassed symptoms like headaches, dizziness, seizures, or weakness suspected to have a neurological basis. The “Other category” included cases that did not fit into existing categories or involved multiple overlapping symptoms without a clear primary system.

#### Discharge Diagnoses

Discharge diagnoses, recorded in the EHR by the treating provider based on history, physical exam, and initial diagnostic evaluation, were categorized into broad diagnostic groups, including infection, cardiovascular, neurologic, and dermatologic conditions, again adapted from the CMS MDCs. Infections were further classified by organ system, including genitourinary, respiratory, dermatologic, ENT, OB/GYN, gastrointestinal, and dental. Other discharge diagnoses categories included musculoskeletal, which encompassed conditions like sprains, fractures, or musculoskeletal pain, psychiatric, neurologic, and other, which included cases that did not fit into a single category or involved multiple overlapping conditions.

#### Emergency Severity Index (ESI) Acuity Level

Nurses assigned ESI acuity levels at triage per standard ER practice based on a five-level triage criterion. This index prioritizes patients according to their clinical stability and the resources they are expected to require. Level 1 includes the most critical patients requiring immediate intervention (e.g., cardiac arrest). Level 2 includes patients at high risk of deteriorating, those with altered mental status, or those in severe pain or distress (e.g., severe chest pain). Levels 3, 4, and 5 are used to differentiate stable patients based on the unique types of resources that will be needed to determine the patient’s disposition. Level 3 includes patients requiring two or more unique resources (e.g., abdominal pain), Level 4 includes patients requiring one unique resource (e.g., ankle pain), and Level 5 includes patients requiring none (e.g., medication refill).

#### Disposition

Disposition refers to the final destination of a patient following their ED visit, categorized as a discharge, admission, transfer, or other relevant outcomes.

#### Preventability of ED Visits

ED visits were classified by the study team according to their prevention status using the NYU Algorithm for Emergency Department Visit Appropriateness (NYU-EDA) [9]. This algorithm classifies ED visits based on whether the visit required emergent treatment in an ED setting and whether they were due to an exacerbation of a preventable chronic condition. Injuries and mental health ED visits are placed in separate categories. Visits are categorized using probabilistic weights applied to discharge diagnoses, which were applied accordingly to this dataset and reported as proportions.

### Statistical Analysis

Descriptive statistics were used to characterize the distribution of ED visit outcomes among patients. Categorical variables were summarized using counts and proportions. Confidence intervals to estimate these proportions were generated using a bootstrap method with resampling at the patient level. This method was chosen to preserve an intra-patient correlation since each patient could have multiple, non-independent ED visits. Patients were randomly resampled with replacement from the sample of 118 individuals. For each resampled dataset, only patients with at least one ED visit were included, and all recorded ED visits for these patients were retained to preserve intra-patient correlation. This process was repeated 1,000 times to generate a distribution of proportions, from which 95% confidence intervals were derived using the 2.5th and 97.5th percentiles of the bootstrap distribution. This approach allows estimation of the expected distribution of health outcomes among the unknown number of patients with ED encounters in the full population of 2,231 patients. All statistical analyses were conducted using R Version 4.4.1.

## Results

The demographic characteristics of these 118 patients with recorded ED encounters are presented in Table 1. Among all patients, there were a total of 319 ED visits, averaging 2.7 visits per person. Most patients were 18 to 39 years old (46%) or 40 to 64 years old (53%), while only 2 patients were 65 years or older (2%). 77 patients were recorded as female (65%) and 41 as male (35%). ED registration recorded most patients’ race as Unknown (60%) or Other (31%). Recorded ethnicity confirmed that the majority, 111 patients (94%), were Hispanic or Latinx. Approximately two-thirds of patients were uninsured (69%), while a quarter had Medicaid (27%), and a small minority had private insurance (3%). Of note, EHR records did not specify if Medicaid referred to temporary Emergency Medicaid (which undocumented people are eligible for) or full-scope Medicaid.

**Table 1:** Demographic characteristics of undocumented patients with ED encounters (*n* = 118). This table provides a summary of the demographic and clinical characteristics of the study population. Categorical variables are presented as frequencies

		Age Group (years)		
Variable	Overall *N* = 118	18–39 *N* = 54	40–64 *N* = 62	65+ *N* = 2
**Sex**				
Female	77 (65%)	37 (69%)	38 (61%)	2 (100%)
Male	41 (35%)	17 (31%)	24 (39%)	0 (0%)
**Race**				
Unknown	71 (60%)	28 (52%)	42 (68%)	1 (50%)
Other	36 (31%)	21 (39%)	14 (23%)	1 (50%)
Native Hawaiian/Pacific Islander	8 (6.8%)	4 (7.4%)	4 (6.5%)	0 (0%)
White	2 (1.7%)	0 (0%)	2 (3.2%)	0 (0%)
Black/African American	1 (0.8%)	1 (1.9%)	0 (0%)	0 (0%)
**Ethnicity**				
Hispanic or Latino	111 (94%)	52 (96%)	57 (92%)	2 (100%)
Unknown	6 (5.1%)	2 (3.7%)	4 (6.5%)	0 (0%)
Non-Hispanic	1 (0.8%)	0 (0%)	1 (1.6%)	0 (0%)
**Insurance Status**				
Uninsured	82 (69%)	36 (67%)	44 (71%)	2 (100%)
Medicaid	32 (27%)	17 (31%)	15 (24%)	0 (0%)
Private Insurance	4 (3.4%)	1 (1.9%)	3 (4.8%)	0 (0%)

**Fig. 2:**
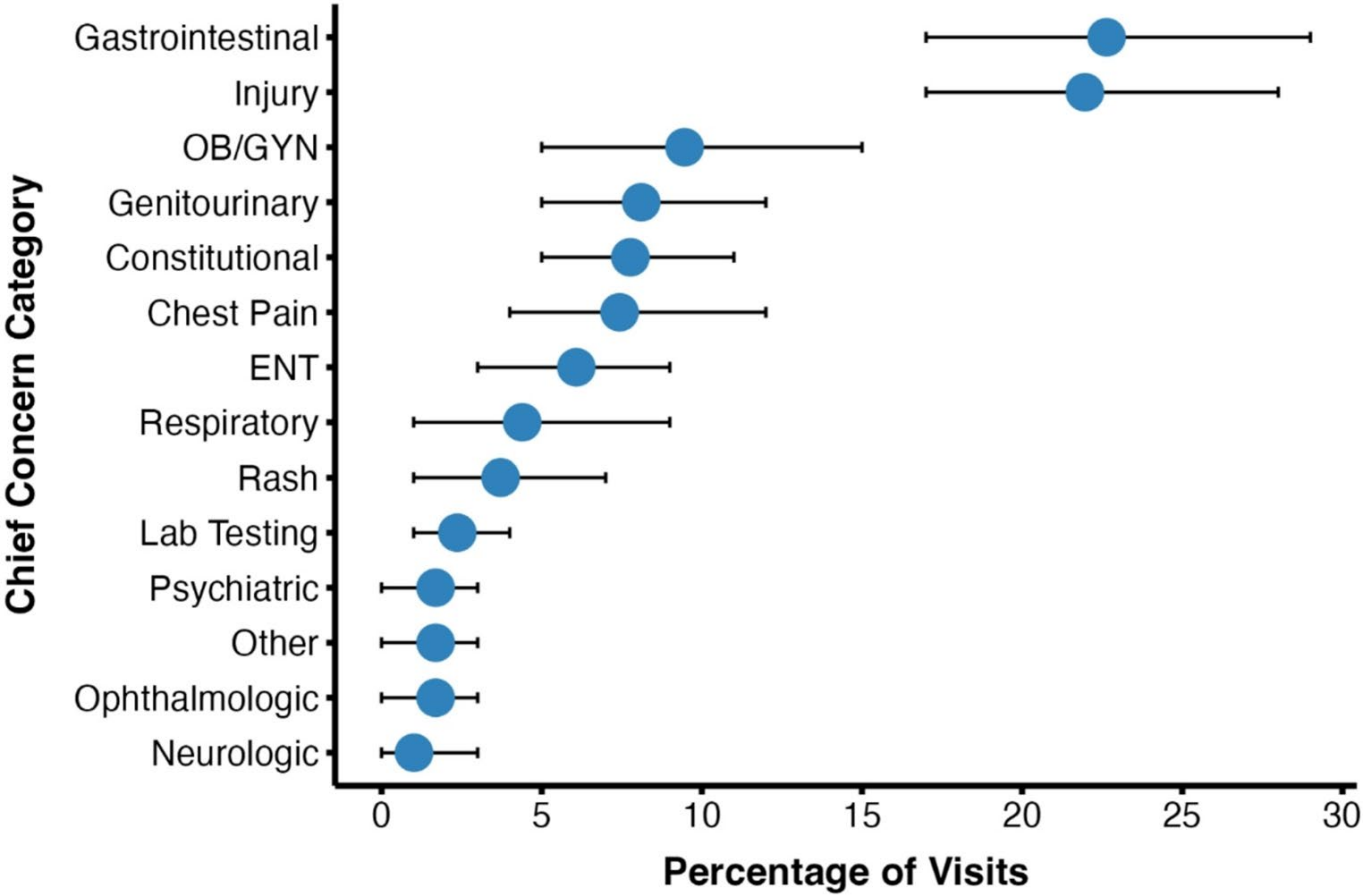
ED visits categorized by chief concerns (*n* = 296, 23 not recorded). ED visits by undocumented patients were categorized into groupings by chief concern. Gastrointestinal concerns and injuries constituted almost half (45%) of all visits. Another quarter of visits were for OB/GYN, genitourinary, or constitutional concerns (25%)

**Fig. 3:**
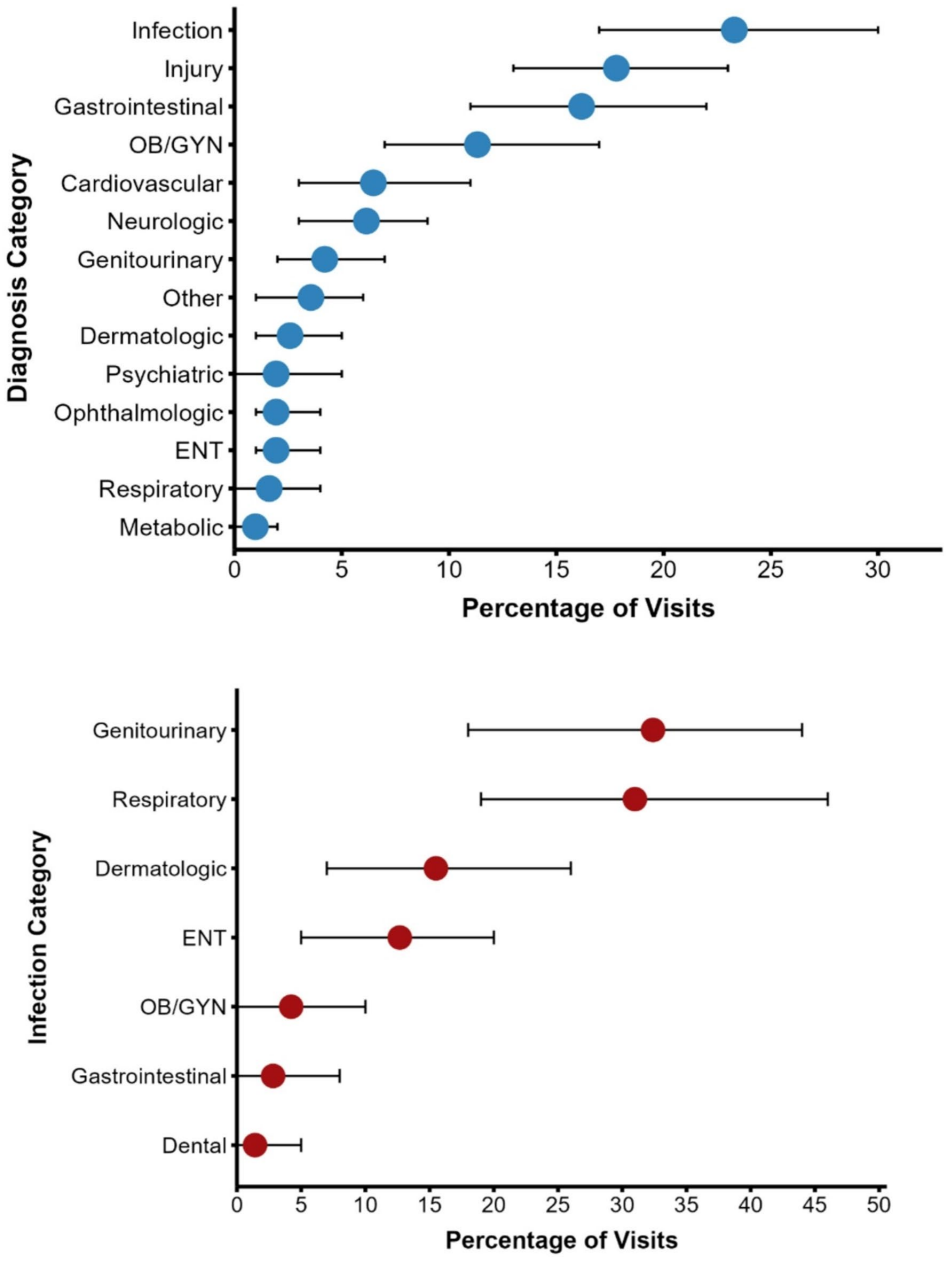
ED visits categorized by discharge diagnosis (*n* = 309, 10 not recorded). ED visits by undocumented patients were categorized into groupings by the diagnosis determined by a clinician. Half of all visits were determined to have an underlying infectious, injurious, or gastrointestinal etiology (57%). Another quarter of visits were determined to be obstetrical/gynecologic (OB/GYN), cardiovascular, or neurologic in origin (23%). The infectious category was further broken down by anatomical source, with genitourinary and respiratory pathogens accounting for two-thirds of all infections (63%)

**Fig. 4:**
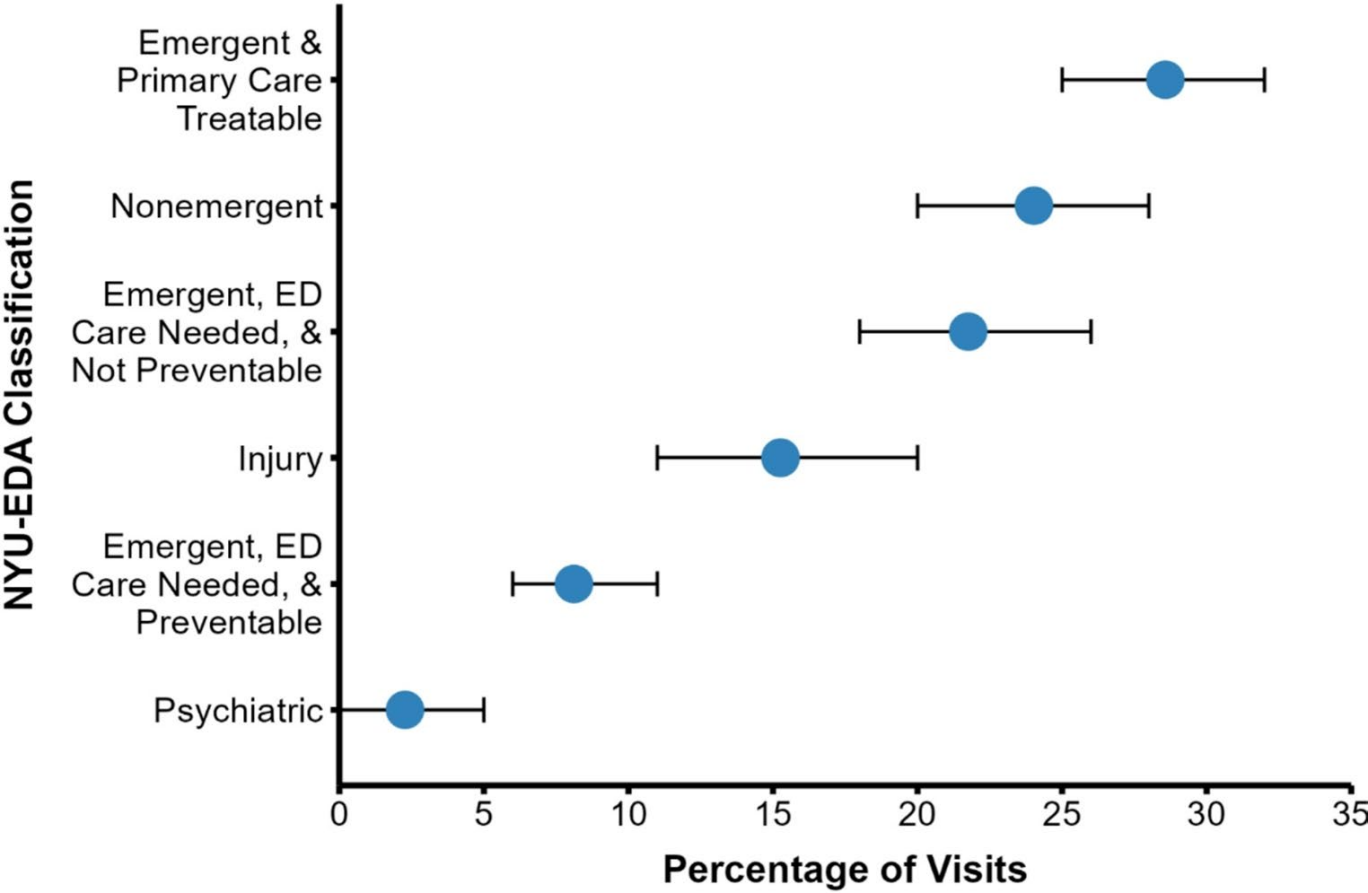
Classification of ED Visits by Prevention Category (*n* = 309, 10 diagnoses not recorded). Similar distributions of ED visits were classified as preventable across age groups. 61% of visits were classified as preventable, primary care treatable, or non-emergent by the NYU ED algorithm (NYU-EDA) criteria

**Fig. 5:**
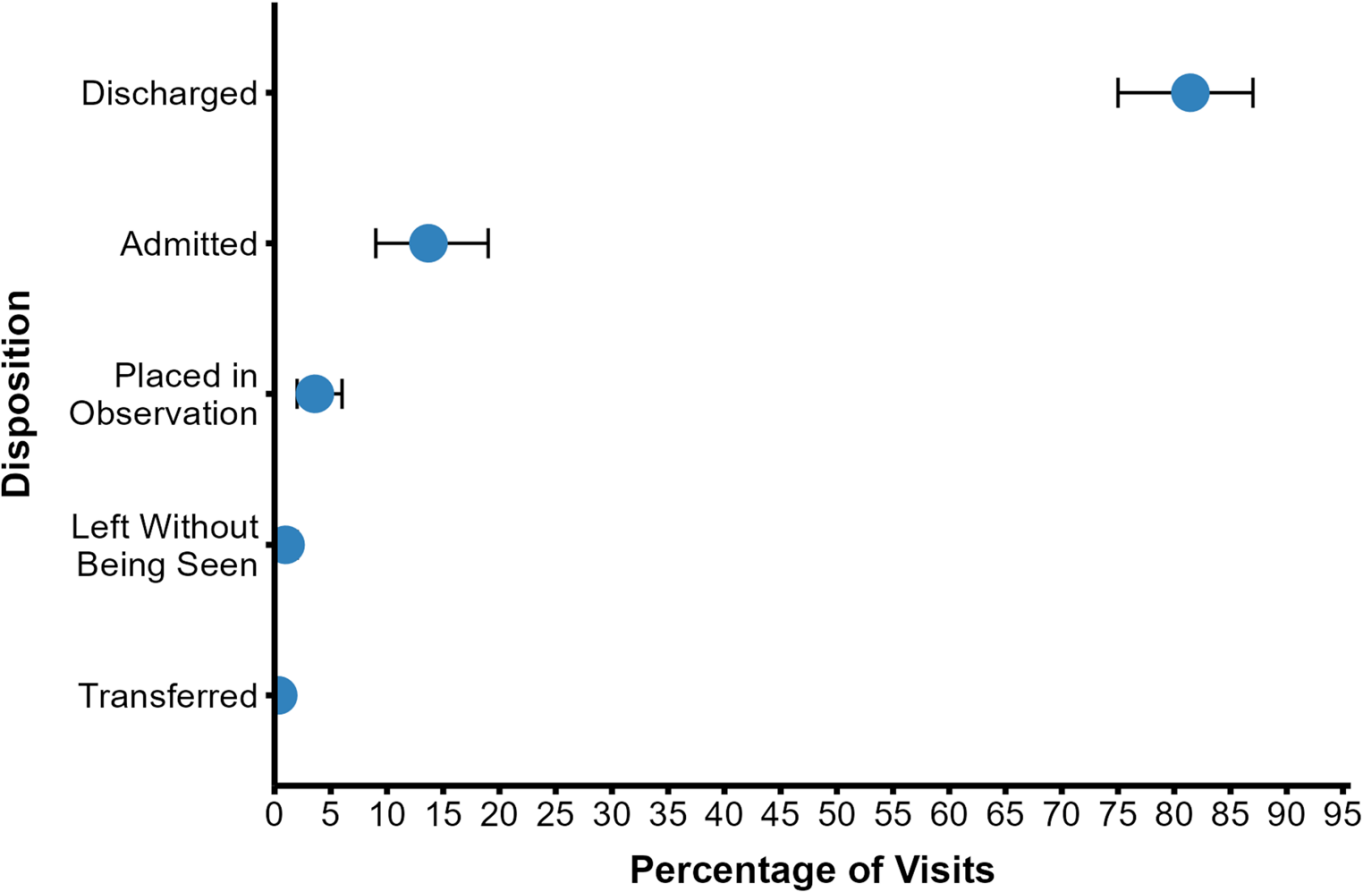
ED Visits by Disposition (*n* = 307, 12 not recorded). A majority of patients (81%) were discharged from the ED. A smaller proportion were admitted (14%) or placed in a 24–48 h observation unit (4%). Few patients left without being seen (1%), and no patients left against medical advice

**Fig. 6:**
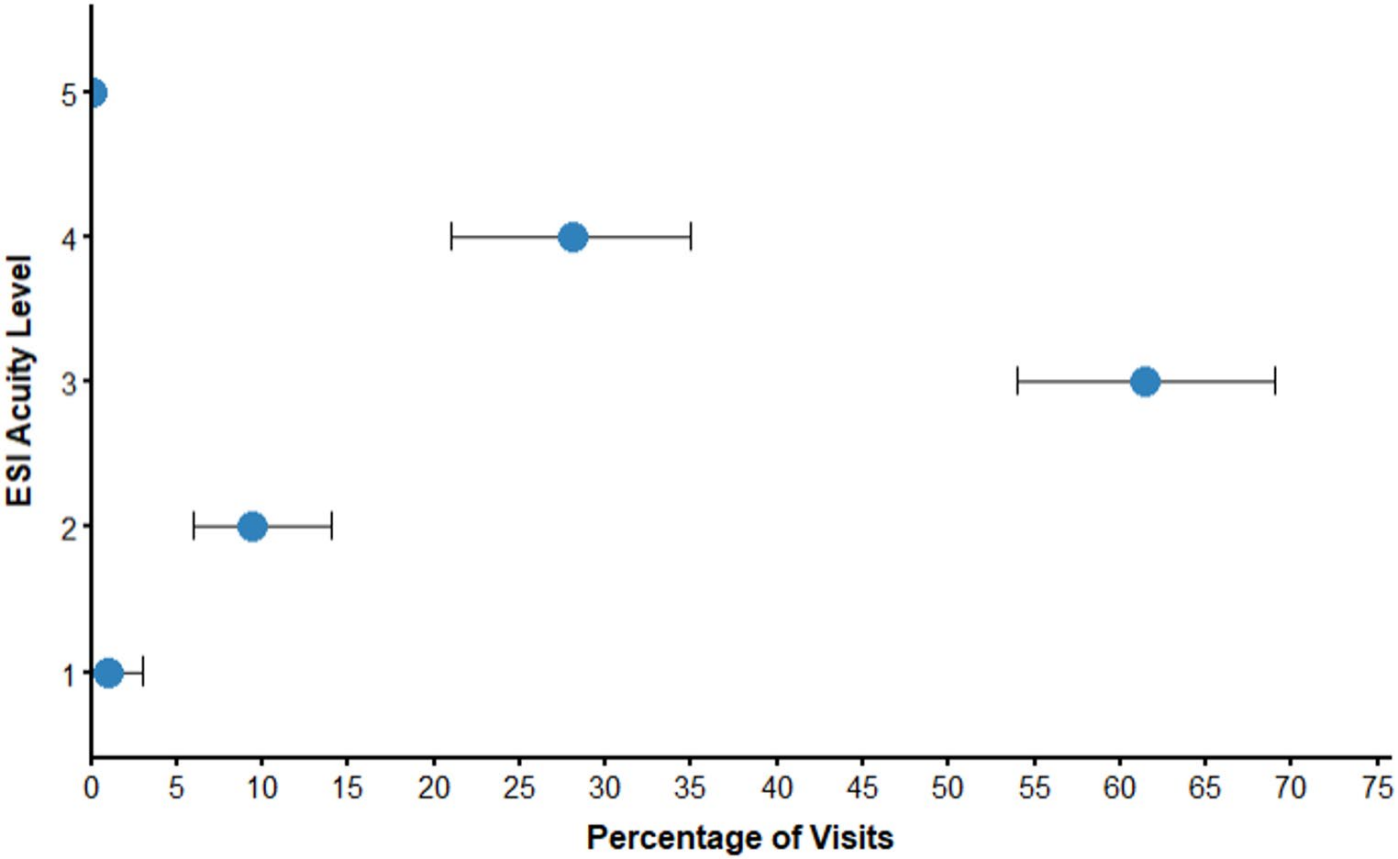
ED Visits by Emergency Severity Index (ESI) Acuity Level (*n* = 192, 127 not recorded). Most visits (89%) were triaged as stable (Levels 3 and 4). Only 1% of visits were triaged as requiring immediate, life-saving intervention (Level 1). 9% of visits were triaged at high risk of deterioration (Level 2). No patients were triaged as nonurgent (Level 5)

​The most common chief concerns leading to ED presentation were categorized as gastrointestinal concerns (23%), injuries (22%), OB/GYN concerns (9%), and genitourinary concerns (8%) (Fig. 2). Regarding diagnosis at discharge, the majority of all visits were determined to have an underlying infectious, injurious, or gastrointestinal etiology (57%) (Fig. 3A). Infectious diagnoses were further broken down by organ system, with genitourinary and respiratory pathogens accounting for two-thirds of all infections (63%) (Fig. 3B). By the NYU ED Algorithm (NYU-EDA) criteria, a quarter of visits (24%) were classified as non-emergent, such as medication refills. Another quarter were emergent and primary care treatable (28%), such as visits for uncomplicated urinary tract infections. A minority were emergent and due to preventable exacerbations of chronic disease (8%), such as asthma exacerbations. Nearly a fourth of visits were classified as emergent and likely not preventable (22%), including acute conditions such as appendicitis. The remaining visits represented injuries (15%), or mental health-related visits (2%) (Fig. 4). A vast majority of ED visits resulted in a direct discharge (81%), few patients left without being seen (1%), and no patients left against medical advice (0%) (Fig. 5). Only 192 visits were triaged according to the Emergency Severity Index (ESI), as not all hospitals documented this. Of these 192 visits, most were triaged as stable levels 3 or 4 (89%) (Fig. 6). Approximately half of the ED visits (48%) were from patients with a single recorded encounter. Many patients had two to five encounters (43%), while a minority had six or more encounters (8%) (Supplementary Figure B). When comparing patients with a single encounter to those with two to five encounters, the distribution of NYU-EDA preventability categories was largely similar. However, the distribution of chief concerns differed— injury-related concerns were more common among patients with a single encounter (30% vs. 21%), while OB/GYN-related concerns were more frequent among patients with multiple encounters (6% vs. 15%).

## Discussion

This study provides valuable insight into the ED utilization patterns of undocumented patients receiving primary care at a CHC in a major US city. The findings reveal a significant proportion of ED visits were classified as preventable or primary care treatable with a high number of low-acuity classifications (ESI Levels 3 and 4). While we recognize that ESI level is an imperfect proxy for patient acuity and that interpretations of acuity can vary by institution, this pattern may reflect underlying gaps in access to timely outpatient care.

Nearly half of all ED visits were attributed to patients with a single recorded encounter. The most common presenting concerns were gastrointestinal issues and injuries, which is consistent with the distribution of national ED visits in the U.S. population. These trends suggest that our sample of undocumented individuals did not utilize ED services any differently than the broader population requiring care in EDs, however further research is warranted to explore these patterns further [9]. Previous research shows that patients with limited English proficiency—like most of our population—are less likely to leave without being seen or against medical advice, supporting our finding that they typically present to the ED for specific acute concerns [10].

In the context of broader literature, our findings suggest that undocumented patients at an urban CHC exhibit ED utilization patterns similar to those observed in the broader CHC population [11, 12]. This challenges assumptions that undocumented status itself is a primary driver of avoidable ED use, instead reinforcing that structural barriers such as limited access to consistent primary care play a more critical role. Additionally, our findings indicate that undocumented patients have similar rates of preventable conditions as defined by the NYU-EDA criteria compared to other CHC populations [13]. This suggests that the distribution of emergent and non-emergent visits among the study population does not markedly differ from national ED utilization patterns, which further supports the idea that undocumented immigrants utilize emergency services in ways similar to other populations. However, this population exhibits a relatively low proportion of visits classified as emergent, requiring ED care, or preventable, a category that reflects exacerbations of chronic disease. This trend may reflect the “healthy immigrant effect,” wherein immigrant populations tend to have lower burdens of chronic illness compared to native-born counterparts, attributed to factors such as the selective migration of healthier individuals and differences in lifestyle behaviors [14].

A large literature review demonstrates challenges in the U.S. with ED utilization and unscheduled care. High volumes of patients present to EDs for various reasons ranging from patient uncertainty about where to seek care and a lack of primary care access [15, 16]. Previous research has shown that CHC patients who are targeted for increased primary care surveillance have a reduction in avoidable ED visits driven by a decrease among high ED utilizers [17]. Our results highlight opportunities for CHCs to improve patient education and guide appropriate care navigation, especially for the most common reasons patients present to the ED. Patients may also benefit from improved outpatient management and care coordination following discharge from the ED to reduce its utilization for non-emergent conditions. Other studies have highlighted that the effectiveness of free clinics in reducing unnecessary ED visits depends on their operational capacity [18]. Clinics with longer hours and a broader range of services were more successful in reducing preventable ED visits compared to those with limited availability, suggesting that expanding service hours and integrating additional specialties may enhance the impact of community health clinics. Expansion of services, while valuable in decreasing ED visits, requires substantial investment of financial and personnel resources, and more work needs to be done to understand the value of such investments by health systems.

The study is limited by its single-site design and relatively small sample size, which may limit the generalizability of the findings to undocumented immigrants across the US and other healthcare settings apart from the ED. Another key limitation is that the NYU-EDA has an estimated accuracy of 53.9% in classifying the medical urgency of ED visits based on discharge diagnoses [13]. This limited accuracy may introduce misclassification bias that would alter the distribution of ED visits across the NYU-EDA prevention categories. Furthermore, discharge diagnoses can also be misclassified for visits with diagnostic uncertainty, incorrect documentation by a provider, or erroneous medical coding. This study was also limited by lack of access to all electronic health records from the area hospitals, likely leading to an underestimation of the true ED utilization in this population. This may also result in selection bias if ED visits at the primary academic health system analyzed in this study differ systematically from ED visits at other nearby health systems. However, this concern is partially mitigated by the integration of 25% of health records from other systems through Care Everywhere (Supplementary Figure C). Additionally, this study’s cross-sectional design limits an analysis of longitudinal healthcare utilization trends, and self-reported barriers to care were not assessed.

While this analysis offers a foundational understanding of ED utilization trends among this population, further research is needed to assess long-term patient outcomes, explore reasons behind care-seeking behaviors, and evaluate the impact of community-based interventions aimed at reducing avoidable ED visits. To further contextualize these findings, future studies should explore comparisons of ED utilization trends across uninsured and insured populations within the same ED settings. Additionally, incorporating qualitative data from patient interviews could provide deeper insights into care-seeking behaviors and the structural barriers faced by undocumented immigrants.

This study provides a crucial first step in understanding ED utilization patterns among undocumented immigrants at CHCs. The findings offer a valuable contribution to the literature on immigrant healthcare utilization and emphasize the importance of improving access to primary care and preventative services. By addressing gaps in care and strengthening primary care services, CHCs can play a pivotal role in improving health outcomes and reducing the strain on emergency departments.

### New Contribution To the Literature

This study fills a gap in the immigrant health literature by describing patterns of emergency healthcare utilization by undocumented populations, leveraging a novel data linkage between a local community health clinic and an academic health system. Our results identify the most common reasons for these ED visits and highlight that a large proportion of the visits are for preventable or primary care treatable conditions. These findings highlight the need for targeted interventions, including injury prevention, improved outpatient management, and expanded CHC services to reduce unnecessary ED visits.

## Electronic Supplementary Material

Below is the link to the electronic supplementary material.

Supplementary Material 1: Supplementary Figure A: Distribution of ED Visits by Order (*n* = 319). About a third of ED visits (37%) in the dataset represented the first ED visit by a patient. About half of ED visits (45%) were 2nd, 3rd, 4th, or 5th visits by a patient. 18% of visits were a 6th visit or more from a single patient. Supplementary Figure B: Distribution of ED Visits by Patient (*n* = 118) About half of ED visits (48%) in the dataset were contributed by patients who had one ED encounter recorded. Another third (43%) of ED visits were contributed by patients who had two to five ED encounters recorded. Patients with 6 or more ED encounters accounted for 8% of the dataset. Supplementary Figure C: ED Visits by Health System (*n* = 316, 3 not recorded) A majority of the ED visits captured (75%) are represented by hospitals in this study’s health system. 14% of visits occurred at a peer health system. Approximately a quarter of visits (25%) were captured through the Care Everywhere system at any hospitals outside this study’s health system.

## Data Availability

Data is provided within the manuscript or supplementary information files. For any additional data requests, please contact the corresponding author.

## Declarations

### Statements and Declarations

The authors have no disclosures.
