# Supplementary material for: Evaluating Emergency Department Utilization among Undocumented Patients Receiving Care at a Community Health Clinic
Source: J Immigr Minor Health. 2025 Jul 10;27(6):906–15. doi: 10.1007/s10903-025-01723-9 (PMC12669307; doi:10.1007/s10903-025-01723-9)
Supplement: Supplementary file 1 — Supplementary Material 1: Supplementary Figure A: Distribution of ED Visits by Order (n = 319). About a third of ED visits (37%) in the dataset represented the first ED visit by a patient. About half of ED visits (45%) were 2nd, 3rd, 4th, or 5th visits by a patient. 18% of visits were a 6th visit or more from a single patient. Supplementary Figure B: Distribution of ED Visits by Patient (n = 118) About half of ED visits (48%) in the dataset were contributed by patients who had one ED encounter recorded. Another third (43%) of ED visits were contributed by patients who had two to five ED encounters recorded. Patients with 6 or more ED encounters accounted for 8% of the dataset. Supplementary Figure C: ED Visits by Health System (n = 316, 3 not recorded) A majority of the ED visits captured (75%) are represented by hospitals in this study’s health system. 14% of visits occurred at a peer health system. Approximately a quarter of visits (25%) were captured through the Care Everywhere system at any hospitals outside this study’s health system. [file 10903_2025_1723_MOESM1_ESM.docx]

**
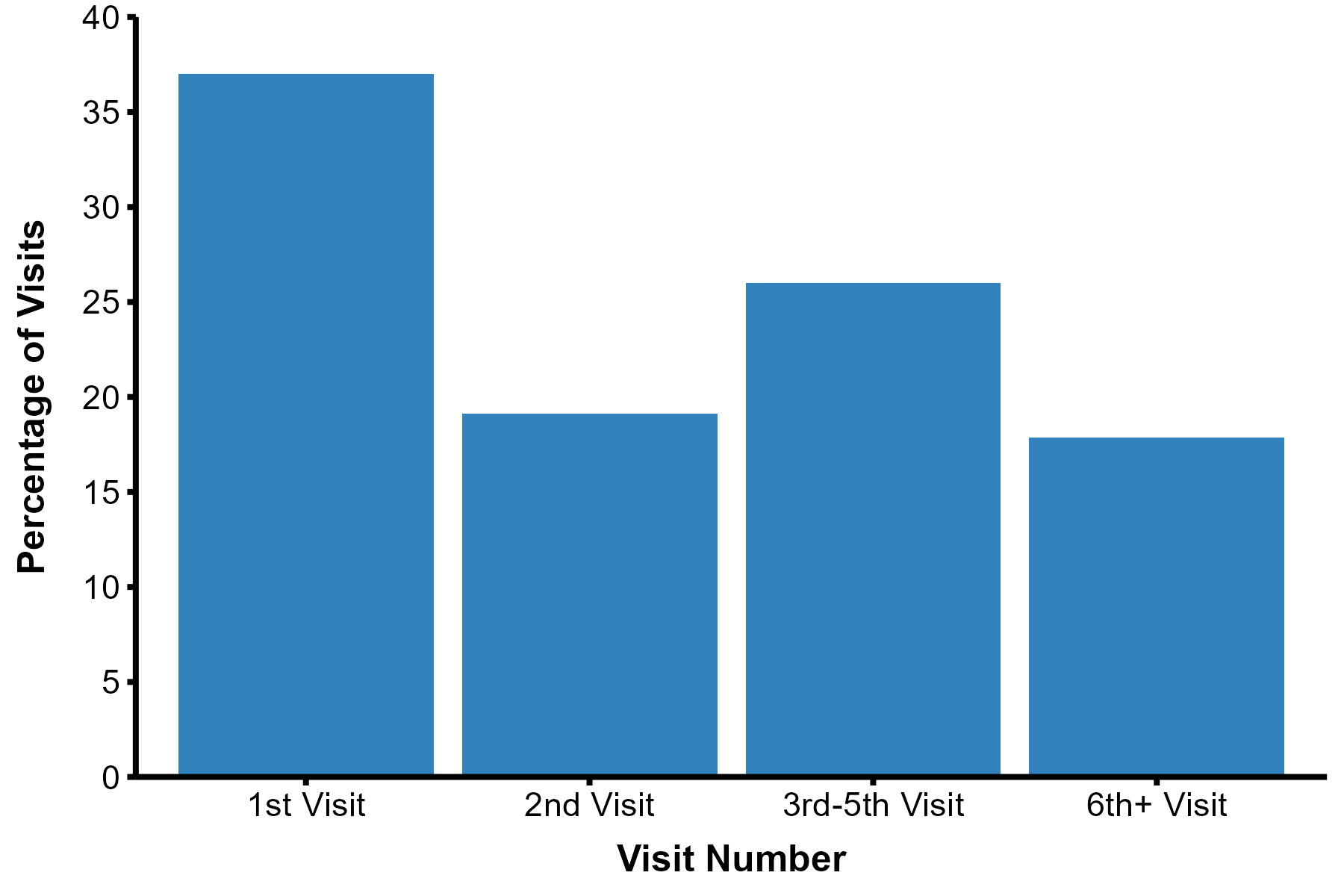
**

**Supplementary Figure A: Distribution of ED Visits by Order (n = 319).** About a third of ED visits (37%) in the dataset represented the first ED visit by a patient. About half of ED visits (45%) were 2nd, 3rd, 4th, or 5th visits by a patient. 18% of visits were a 6th visit or more from a single patient.


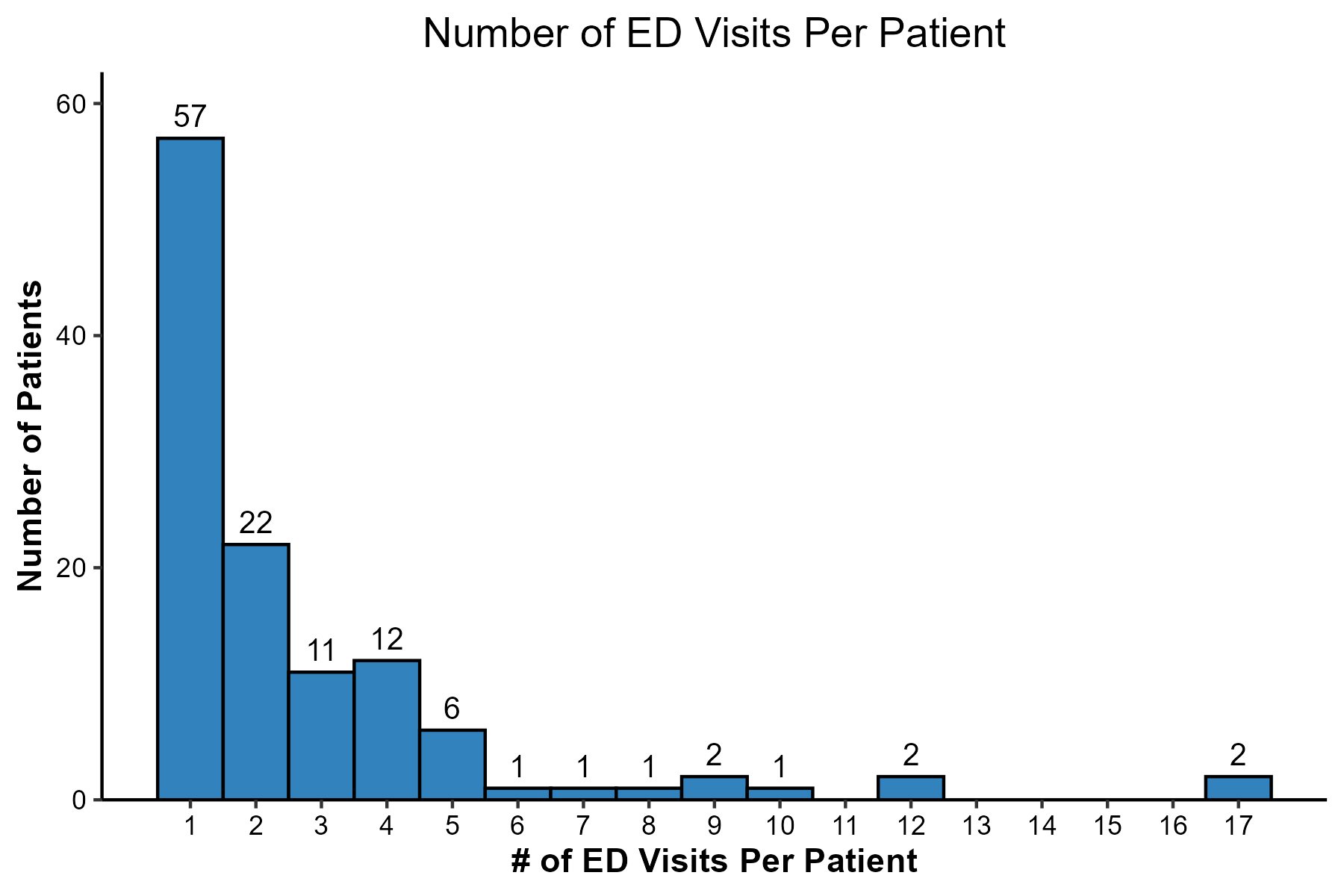


**Supplementary Figure B: Distribution of ED Visits by Patient (n = 118).** About half of ED visits (48%) in the dataset were contributed by patients who had one ED encounter recorded. Another third (43%) of ED visits were contributed by patients who had two to five ED encounters recorded. Patients with 6 or more ED encounters accounted for 8% of the dataset.


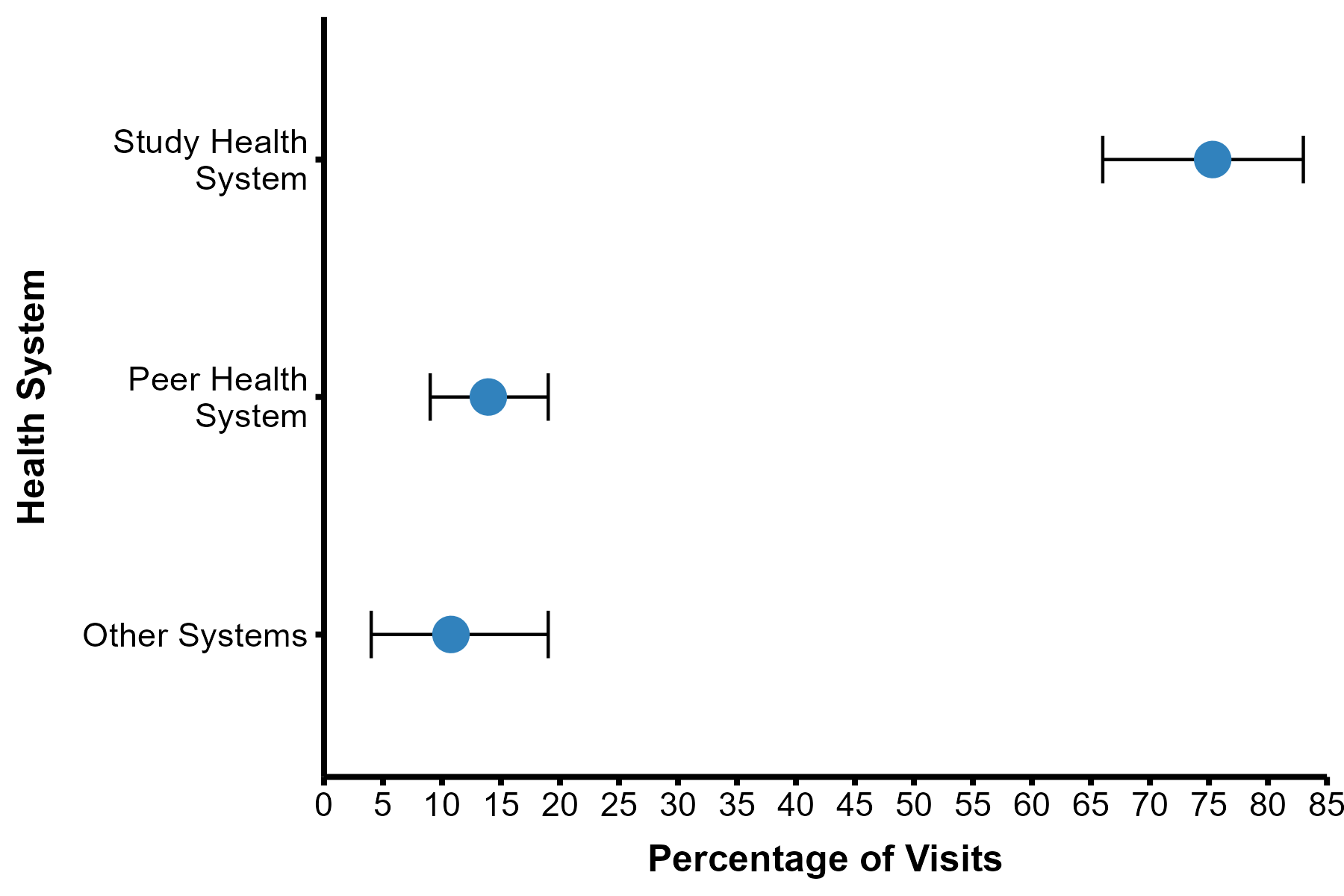


**Supplementary Figure C: ED Visits by Health System (n = 316, 3 not recorded).** A majority of the ED visits captured (75%) are represented by hospitals in this study’s health system. 14% of visits occurred at a peer health system. Approximately a quarter of visits (25%) were captured through the Care Everywhere system at any hospitals outside this study’s health system.
